# A Unified Framework for Brain Segmentation in MR Images

**DOI:** 10.1155/2015/829893

**Published:** 2015-05-18

**Authors:** S. Yazdani, R. Yusof, A. Karimian, A. H. Riazi, M. Bennamoun

**Affiliations:** ^1^Malaysia-Japan International Institute of Technology (MJIIT), Universiti Teknologi Malaysia, 54100 Jalan Semarak, Kuala Lumpur, Malaysia; ^2^Department of Biomedical Engineering, Faculty of Engineering, University of Isfahan, Isfahan 81745, Iran; ^3^Control and Intelligent Processing Center of Excellence, School of Electrical and Computer Engineering, University College of Engineering, University of Tehran, Tehran 14174, Iran; ^4^School of Computer Science and Software Engineering, The University of Western Australia, Perth, WA 6907, Australia

## Abstract

Brain MRI segmentation is an important issue for discovering the brain structure and diagnosis of subtle anatomical changes in different brain diseases. However, due to several artifacts brain tissue segmentation remains a challenging task. The aim of this paper is to improve the automatic segmentation of brain into gray matter, white matter, and cerebrospinal fluid in magnetic resonance images (MRI). We proposed an automatic hybrid image segmentation method that integrates the modified statistical expectation-maximization (EM) method and the spatial information combined with support vector machine (SVM). The combined method has more accurate results than what can be achieved with its individual techniques that is demonstrated through experiments on both real data and simulated images. Experiments are carried out on both synthetic and real MRI. The results of proposed technique are evaluated against manual segmentation results and other methods based on real T1-weighted scans from Internet Brain Segmentation Repository (IBSR) and simulated images from BrainWeb. The Kappa index is calculated to assess the performance of the proposed framework relative to the ground truth and expert segmentations. The results demonstrate that the proposed combined method has satisfactory results on both simulated MRI and real brain datasets.

## 1. Introduction

Visualization and three-dimensional (3D) processing of medical images are rapidly growing fields of study. In particular accurate and robust technique for image segmentation is a research topic which has been one of the core problems in medical image analysis for years. In particular, the segmentation of brain MR images aiming to assign each voxel to a specific tissue class has received considerable attention.

Multimodality imaging techniques are valuable to medical and clinical studies, as well as other fields. Positron emission tomography [1], computed tomography (CT), magnetic resonance imaging (MRI), digital mammography, ultrasound or single photon emission computed tomography (SPECT), and X-ray provide effective ways for the representation of the subject's anatomy [2, 3]. High spatial resolution and good soft-tissue contrast in MR brain images and also recent progress in MRI systems make them suitable for the realization of this goal [4, 5]. In medical and clinical research on brain structures, the description of tissue size is an important aspect of therapy that should be performed accurately.

Most procedures rely on a slice-by-slice interactive input of human knowledge that is very labor intensive and time consuming. These methods suffer from inter- and intraobserver variability [4]. Intraobserver variability occurs when the same users make various choices on different occasions, producing different results each time [6, 7]. Interobserver variation occurs when different users make different selections, which affect the segmentation results [8]. This generally leads to the need for reliable and accurate automatic segmentation of MRI brain images and also to define tumors or lesions if present [9, 10].

In addition, a robust segmentation of lesions is a very important stage for diagnosing disease [11, 12], monitoring treatment, investigating disease progress, and computer-integrated surgery. It is also of noticeable interest to study regional volumes of white matter (WM) and gray matter [13] across several developmental stages of the brain [5, 14, 15]. In the context of neuroimaging, automatic three-dimensional (3D) segmentation of brain MR images into WM, GM, and cerebrospinal fluid (CSF) has received an enormous amount of attention, as it is extremely important for quantitative analysis of MR images. In this paper, we used some techniques for brain segmentation into WM, GM, and CSF.

There has been a wide range of automatic segmentation techniques proposed in the literature. The main problems found in the automatic segmentation of MR images derive from the fact that the intensities of images are not necessarily constant for each tissue class [16].

Among fuzzy clustering algorithms, Fuzzy *C*-Mean is a powerful technique that has been extensively used in MR image segmentation [17] in which voxels are partially classified into various classes using different memberships for each class [18, 19]. Fuzzy *C*-Mean was first conceived by Dunn [20] and generalizes *K*-means algorithm to allow soft segmentation [21]. Pham extended the standard FCM technique to deal with brain MR images corrupted by bias field. The greatest drawback of FCM is its sensitivity to noise. MR images always include a considerable amount of noise, leading to further degradation with segmentation. Many extensions of FCM have been reported in the literature to overcome its drawbacks, but most of them still have some problems [22].

Many researchers segmented brain MRI by applying an artificial neural network (ANN). In comparison with FCM, the FCM algorithm was shown to be worse for abnormal brain with tumor, edema, and so forth and superior on normal brain.

Lemieux et al. have segmented brain MR images into WM, GM, and CSF using Gaussian mixtures and morphological operations [5, 23]. Homomorphic filtering techniques for eliminating the effect of the bias field have been commonly used because of their easy implementation. The problem is that this method is effective only on low contrast images and some researchers reported undesirable artifacts with this method.

One of the main drawbacks of classifiers and clusters is that they do not contain contextual information. The classification of a voxel is quite independent of all the other voxels. One solution for dealing with this problem is applying Markov random fields (MRFs), which is a statistical model in the group of random field methods. In the literature, 3D MRF has been used for tissue classification, which assumes a Gibbs prior to the Gaussian mixtures. It is equivalent to a Gibbs joint probability distribution, which is defined by an energy function [5, 24].

Generally classification techniques dealing with MR images can be divided into two categories: parametric and nonparametric methods. The parametric approaches usually make the assumption that the tissues of brain follow a Gaussian distribution. The statistical model parameters usually are estimated applying a maximum a posteriori (MAP), maximum likelihood method and the expectation–maximization (EM) algorithm that is used for the optimization process [25, 26].

An iterative algorithm based on the EM method algorithm was proposed by Wells III et al. [27]. The algorithm is also designed for eliminating anatomical features of the image, along with intensity nonuniformity field estimations. The advantages of the EM algorithm are its ease of implementation, conceptual simplicity, and also the fact that each of the iterations improves the results. A main problem of EM method is that it is based on a Gaussian distribution model for the intensity distribution of images, which is not true, especially for noisy MRI. In this paper, we proposed a modified EM method as an initial segmentation stage. The proposed EM algorithm overcomes the shortcoming of the standard EM technique using asymmetric Gaussian.

In addition machine learning algorithms have proven to yield desirable results in many cases. The SVM method is considered as a good candidate because of its high generalization performance without the need of prior knowledge, even when the dimension of the input space is very high [28]. The SVM was first proposed by Vapnik and has since attracted a high degree of interest within the research community in the category of machine learning. Some papers have reported that the SVM generally is more able to deliver higher performance in terms of classification accuracy than the other classification techniques as SVMs do not suffer the limitations of limited samples and data dimensionality [29]. In our study, support vectors, which are critical for classification, are created by learning from the training samples, which are extracted from the previous stages.

The key aspect of the proposed automatic framework is that we divided the segmentation task into three stages, each of which extracts a different set of constraints of the problem, and also the output of each stage simplifies the one which follows it. The first step of our method after preprocessing steps falls into the category of statistical segmentation techniques and provides an intensity-based classification. In this stage we modified EM algorithm for modeling the underlying distributions of WM, GM, and CSF, which is an initial classification step. Then we extracted textural features from the target areas that include both nonoverlapped and overlapped voxels. By applying feature extraction method based on cooccurrence features prior to SVM algorithm, a reliable class labeling for the image will be generated, thereby facilitating the SVM step. Subsequently, we used SVM for margin classification and segmentation enhancement. The goal of using SVM is to assign a label to each overlapped voxel of the brain borders and to enhance the segmentation result.

It is demonstrated that a robust and accurate segmentation approach can be achieved to find optimal segmentations. That is demonstrated through experiments on both real data and simulated images. The rest of this paper is organized as follows.

In Section 2 we present the new automatic method for segmentation of brain tissues that combines three techniques with some new ideas and that is more robust than its individual components. We give a step-by-step explanation for estimating model parameters. In Section 3, we present experimental results of the proposed technique. In this section we discuss issues regarding verification of medical image segmentation and also present a comparison of our results on simulated and real database. The segmentation performance is evaluated for the proposed method.  Section 4 contains discussion and concluding remarks.

The proposed combination method is an accurate and fast way to find optimal segmentations, given the intensity models which incorporate the spatial coherence assumptions.

## 2. Materials and Methods

Our classification method involves three steps: modified EM based segmentation method, feature extraction, and nonlinear classification, which are detailed next. In the first step before brain classification we attempted to extract the brain from MR images. To compensate for the inhomogeneity and partial volume effects the preprocessing steps are applied prior to actual segmentation, which are explained as follows.

### 2.1. Skull Stripping

The first task for MRI analysis is to define brain and nonbrain voxels. This work is concerned with the predominant brain tissues: WM, GM, and CSF. The measured signal intensities of these predominant tissues may overlap other tissues, such as bone, fat, skin, dura, and muscle. This problem complicates reliable brain segmentation. Brain surface skull stripping is one of the important preprocessing steps for MRI segmentation. In this paper, we removed the skull, scalp, and other extraneous tissues of brain images by using the Brain Surface Extractor (BSE).

### 2.2. Image Nonuniformity Compensation

The magnetic susceptibility variations in the MR images cause the intensity nonuniformities (bias field) that prevent description of voxel tissue content based exclusively on image intensity [30]. Consequently segmentation and quantitative analyses of MR images require bias field correction. We applied the Bias Field Corrector (BFC) software to each of the images after skull stripping with BSE. The BFC is utilized to compensate for the intensity nonuniformity [26]. Both BSE and BFC are implemented in BrainSuite package (http://brainsuite.usc.edu/).

### 2.3. Partial Volume Estimation

Partial volume estimation (PVE) is caused by the finite spatial resolution of imaging devices, due to the complexity of human brain anatomy. This phenomenon is created in MR images when more than one tissue type occurs in a voxel, and a voxel is a combination of different tissues, such as WM and GM [27, 28]. The partial volume effect blurs the intensity distinction between tissue classes at the edges of the two tissues. The estimation of the amount of each tissue type within each voxel has received considerable interest in recent years. The PVE is an important stage when a robust and accurate segmentation is needed.

We used the trimmed minimum covariance determinant (TMCD) technique for the estimation of the parameters of the PV model in this paper [31]. This technique is based on trimmed minimum covariance determinant parameter estimation and MRF based tissue classification [29]. We computed the fractional tissue values for each image using the PVC software.

The results are three images of the three primary tissue types of CSF, GM, and WM, respectively, whose elements reflect the proportion of the corresponding tissue type in each voxel. Also the algorithm classifies the voxels into the three primary tissue types and their partial volume mixtures (CSF/background, CSF/GM, and GM/WM). According to the partial volume classification, the voxels belonging to the pure CSF or CSF/background are removed from the skull stripped volume and the remaining volume will be processed for decomposition.

### 2.4. Registration and Atlas Alignment

In the preprocessing step we performed a spatial registration (alignment) of the input images. Image registration is the operation of aligning images to relate corresponding features. For most kinds of image processing on two or more images, it is required that the images are aligned, so that one voxel position represents the same anatomical position in all images. We performed affine registration with 12 degrees of freedom [30–32].

### 2.5. Brain Segmentation

In this paper, a novel algorithmic framework is proposed, in which we integrated different types of information, MR intensity, textural features, voxel location, and relationship with neighboring voxels, to improve the overall segmentation performance.

The first step is extended EM algorithm to initially segment the brain into three tissues. The results of modified EM for initial segmentation are superior to standard EM. The reasons that we combine modified EM to the next stages are as follows.

- Since the voxels of brain regions, especially at the edges and borders, are not defined by unique intensities in MR images due to the presence of artifacts and overlapped voxels, further processing is also needed to ensure robust segmentation. Due to mentioned problems, some of the voxels that have been segmented in the first stage have two tissue types such as GM, CSF or GM, WM.
- Since the intensity information which is used in the first step is not sufficient to have powerful segmentation, textural features and spatial relationships of voxels are investigated in the next steps.

**Figure 1:**
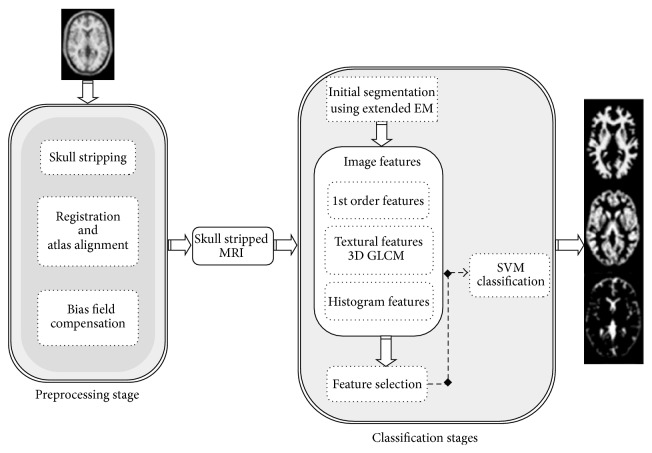
General overview of the proposed technique.

In the second step we extracted some textural features of nonoverlapped regions and also to improve the SVM training process, some features of overlapped voxels were extracted randomly. Finally SVM algorithm is applied to identify overlapped voxels using extracted features. We also used SVM classifier for the brain margin classification and segmentation enhancement. In other words in the SVM stage we applied nonoverlapped and overlapped voxels for training stage and overlapped voxels for testing stage. Using overlap voxels in addition to nonoverlapped regions for SVM training step leads to more accurate segmentation. Therefore the SVM stage improves the segmentation results. It represents the results of each method which are then refined with the next method. In the next section three steps are described. A general overview of our method is shown in Figure 1.

### 2.6. EM-Based Algorithm

The expectation-maximization algorithm (EM) is an algorithm to find missing data based on observed data and maximum likelihood parameter estimates. In automated model-based bias field correction of MR images of the brain the observed information is the intensities of the image, the missing data are the labels, and the parameters are the standard deviations and means of the Gaussian distribution, which is assumed for the intensity distribution of each tissue class. The EM algorithm is an iterative method, which interleaves two steps: the expectation step (E-step), which is the computation of posterior probabilities of each voxel belonging to each class (WM, GM, and CSF), and the maximization step with maximum likelihood estimation of the Gaussian distribution parameters. The maximum values are then taken as the new parameters [32].

The random observations are the intensity of the *n*th voxel in a brain region of interest [33]. Let *θ*_*i*_ = {*θ*_1_,…, *θ*_*k*_} and *θ*_*i*_ = (*μi*, *σ*_*i*_^2^) demonstrate the parameters of Gaussian distributions. The Gaussian mixtures can be expressed to denote the parameters of *K* Gaussian distributions. The Gaussian mixtures of *K* tissue classes can be explained as

(1) $$\begin{array}{c} p\left(y_{n}\;|\;\omega ,\theta \right)=\overset{k}{\underset{i=1}{\sum }}\omega_{i}\cdot p\left(y_{n}\;|\;\theta_{i}\right), \end{array}$$

where *ω* = {*ω*_*i*_∣0 ≤ *ω*_*i*_, ∑_*i*_ *ω*_*i*_ = 1, *i* = 1,…, *K*} are the weights of tissue classes, and

(2) $$\begin{array}{c} p\left(y_{n}|\theta_{i}\right)=\frac{1}{\sqrt{2\pi }\sigma_{i}}\exp\left\{-\frac{{\left(y_{n}-\mu_{i}\right)}^{2}}{2\sigma_{i}^{2}}\right\},\\ \omega =\left\{\omega_{1},\ldots ,\omega_{k}\right\},\;\theta =\left\{\theta_{1},\ldots ,\theta_{k}\right\},\\ y=\left\{yn|n=1,\ldots ,N\right\}. \end{array}$$

The unknown parameters (*ω*, *θ*) can be estimated applying the maximum likelihood estimation (MLE) technique. *N* is the number of voxels, and *y* is the set of voxel intensities. The likelihood of the voxel intensity data with unknown parameters is shown as follows:

(3) $$\begin{array}{c} L\left(y\;|\;\omega ,\theta \right)\equiv \overset{N}{\underset{n=1}{\prod }}p\left(y_{n}\;|\;\omega ,\theta \right)=\overset{N}{\underset{n=1}{\prod }}\left\{\overset{K}{\underset{i=1}{\sum }}\omega_{i}p\left(y_{n}\;|\;\theta_{i}\right)\right\}. \end{array}$$

The ML estimates are created by solving for the parameters in the normal equations, which are derived from first partial derivatives of (3) that are equated to zero with respect to the unknown parameters (*ω*, *θ*). The same notations are applied for the conditional probabilities and their estimates [4, 5].

Let *p*(*i*∣*y*_*n*_) be the posterior probability and the random observation belongs to the *i*th category. Consider

(4) $$\begin{array}{c} p\left(i\;|\;y_{n},\omega^{\left(t\right)},\theta^{\left(t\right)}\right)=\frac{\omega_{i}^{\left(t\right)}p\left(y_{n}\;|\;\theta_{i}^{\left(t\right)}\right)}{\sum_{j}^{}\omega_{j}^{\left(t\right)}p\left(y_{n}\;|\;\theta_{j}^{\left(t\right)}\right)}. \end{array}$$

The expectation step updates the posterior probability given the latest estimates of unknown parameters (*ω*, *θ*) which is in the *t*th iteration. The likelihood equations admit the posterior probability *p*(*i*∣*yn*, *ω* ^(*t*)^, *θ* ^(*t*)^). The maximization step estimates *ω* and *θ* by inserting (4) into these equations [7]:

(5) ωi(t+1)=∑np(i ∣ yn,ωt,θt)∑j∑np(j ∣ yn,ωt,θt),μi(t+1)=∑np(i ∣ yn,ωt,θt)·yn∑np(i ∣ yn,ωt,θt),σi2(t+1)=∑np(i ∣ yn,ωt,θt)·yn−μi(t+1)2∑np(i ∣ yn,ωt,θt),

where *μ*_*i*_ and *σ*_*i*_ are the mean intensity and the standard deviation of the *i*th class, respectively. The algorithm simply continues iterating between E-step and M-step until *θ* ^(*t*)^ convergence to local maxima.

The segmentation result is then obtained by allocating the *n*th voxel to its tissue class with the maximum posterior probability in (4) [4, 5].

### 2.7. Modified EM

**Figure 2:**
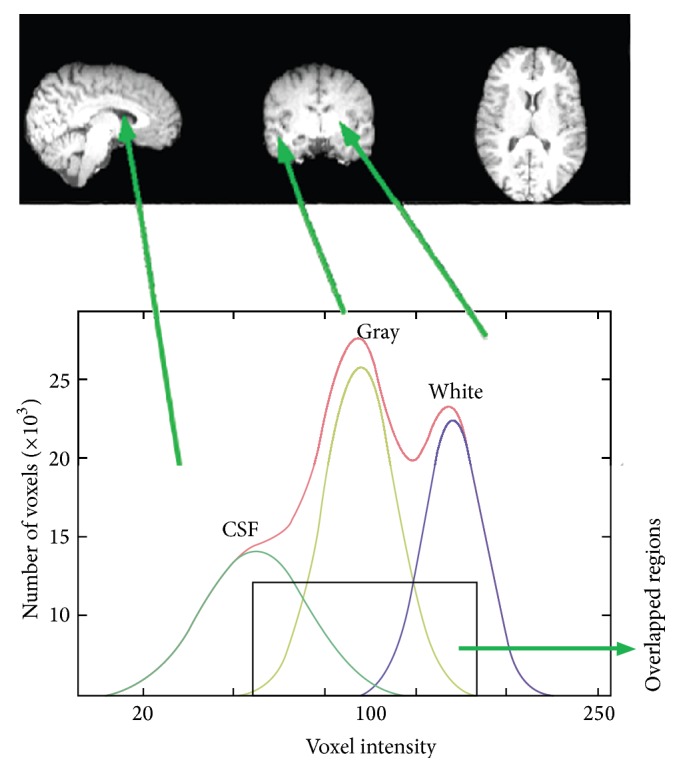
Illustration of symmetric Gaussian distribution and overlapped regions in the histogram.

Image classification based on the EM method essentially models the intensities of an image as a finite mixture of *K* tissue classes. The classification based on standard EM may not recognize individual tissue types accurately. In other words the main shortcoming of the EM based techniques is that they are based on symmetric Gaussian distribution model for the intensity distribution of brain images (see Figure 2). That is not true in the real MRI, especially for noisy images. In real images the estimated Gaussian distribution is not well fitted by the original histogram [5] because they do not have three normal Gaussians due to the existence of noise, artifacts, and overlapped Gaussians in the histogram (Figure 2).

**Figure 3:**
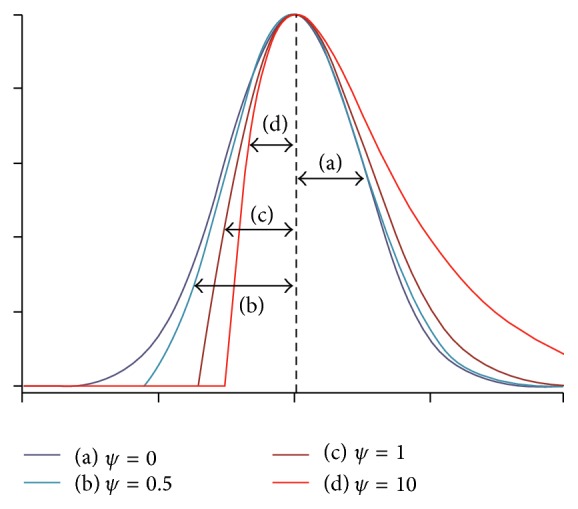
Skewed shapes of asymmetric distributions.

Usually in real MR images the standard deviations of two sides of estimated Gaussian are different from each other. Thus the intensity distributions of brain tissues can vary asymmetrically in these images. Consequently the intensity of individual tissues may display skewed or spread shapes between brain images that may not be well fitted by a Gaussian distribution (Figure 3). For example, the CSF intensity on T1 brain images usually spreads across a wide range at the lower end of the histogram and displays an overlap with the GM tissue [5]. In this kind of asymmetric Gaussian distribution, if we use standard EM, the estimated standard deviation of one side is true and the other side is not accurate. Therefore the estimation of parameters is not completely acceptable.

Extended EM using asymmetric distribution is explained in detail as follows.

As mentioned before, EM algorithm is used to estimate mean and standard deviation of Gaussian distribution in two different steps (E-step and M-step) to reach the optimum Gaussian model. In the extended model, the asymmetric Gaussian has observation variable *x*, latent variable *z*  (*z* ∈ *R* ^*d*^), and the orthonormal matrix *ϕ* ∈ *R* ^*d*×*d*^ : *x* = *ϕ*_*z*_ [34]. The latent variable is a different point between asymmetric Gaussian and standard EM method. The following distribution defines probability density functions for each *z* element. Consider

**Figure 4:**
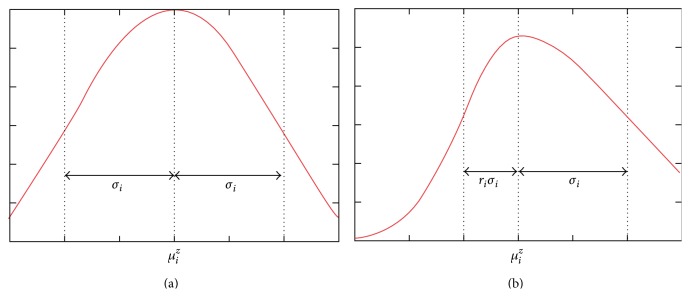
From (a) to (b) symmetric estimated Gaussian and asymmetric Gaussian of real image.

(6) Azi;μiz,σi2,ri≡22π1σi2(ri+1) ·exp⁡⁡−zi−μiz22σi2if  zi>μiz,exp⁡−zi−μiz22ri2σi2otherwise,

where *μ*_*i*_ and *σ*_*i*_ are estimated using standard EM and *r*_*i*_ is the linear coefficient between the standard deviation of two sides. Equation (6) demonstrates that the mentioned density model has an asymmetric distribution (see Figure 4). In this study, we suppose that the *μ*_*i*_ and *σ*_*i*_ are the mean and standard deviation of one side of Gaussian, respectively, and the relationship between two standard deviations is as follows:

(7) $$\begin{array}{c} \sigma_{\text{second-side}}=r\cdot \sigma_{\text{first-side}}. \end{array}$$

**Figure 5:**
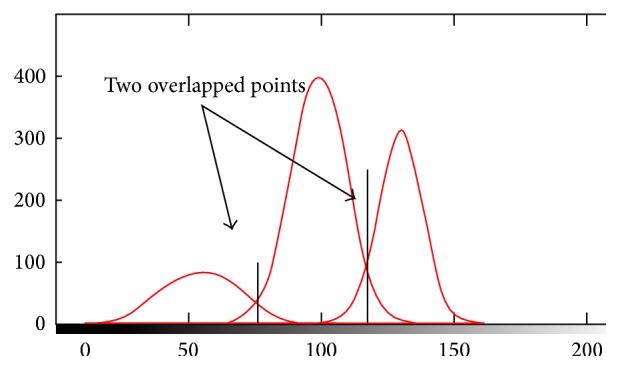
Illustration of two overlapped point.

As mentioned before brain MR images have a mixture of three Gaussian distributions (7), each of which is estimated separately in this study. To compute the error, we separate each distribution in the histogram. Therefore, before error estimation we simply split the whole histogram into three separate Gaussians. These three Gaussians have overlapped at two points, in which sign of the gradient is changed and these two points are also local minimum (see Figure 5). It means that concavity at these two points is positive. Thus the overlapped points are the points in which the sign of first derivative is changed and sign of the second derivative is positive. This is an easy gradient-based method to separate three Gaussian distributions. In this step, we have two overlapped points in the histogram that give three distributions or three classes, which are WM, GM, and CSF.

In the next step, for each Gaussian, we fixed the estimated *μ*_*i*_ and *σ*_first-side_ as the final value and then we determined the estimated standard deviation of the other side. To define the final value of *σ*_second-side_, we applied an iterative error-based technique between the estimated Gaussian and real image histogram. Since we assumed that the distribution is (6), the standard deviation of one side of each Gaussian is proportional to the standard deviation of the other side (see (7)). In the next step, we supposed that the initial value of *r* is 1 (*r* = 1); then *r* value is increased progressively. “*r*” value should be increased step by step. The increasing trend of “*r*” will be continued till the error reaches the threshold value. Experimentally this step is fixed to 10%. Smaller steps increase the processing time and accuracy but the percentage of increased accuracy is not significant. We defined the threshold value using RMS threshold method [35].

The error is calculated for each *r* that is a natural or real number. If error is increased dramatically by increasing *r*, the amount of *r* should be decreased from the initial value. If increasing *r* reduces the amount of error this process will be continued till the error has reached the threshold value (minimum error). The minimum calculated error demonstrates the best value of *r* and consequently the final value of standard deviation.

However the method requires a large number of iterations to reach the defined threshold or convergence. In other words the algorithm starts with some initial value of the parameters, one cycle between the E- and M-steps until *θ* ^(*t*)^ converges to a local maxima. In theory, the EM based methods are guaranteed to converge and they perform a ML estimation of the model parameters at a fast convergence rate. To accelerate the convergence rate of our algorithm, we provide a stopping criterion using the RMS error [35]. By applying consecutive RMS errors we are able to find when to progress or stop the procedure [35].

The minimum error is calculated for three tissue classes. In other words, based on the estimated error, we changed the standard deviation to obtain the best value. This is an iterative technique to reach the optimum parameters.

To compute the error, we considered all intensities of brain MR images. In other words, the estimated distribution and real image histogram should be compared at each intensity. The amount of error is the average difference between estimated distribution and original distribution at related intensity:

(8) $$\begin{array}{c} \text{Error}=\frac{1}{N}\overset{N}{\underset{i=1}{\sum }}\left(f_{i}-g_{i}\right), \end{array}$$

where *N* is the number of intensities that consists of Gaussian distribution, *f*_*i*_ is the number of voxels in related intensity of real histogram, and *g*_*i*_ is the number of voxels in related intensity of estimated histogram. Consequently, in this section minimum error and optimum parameters are estimated. Finally, it is repeated two more times to provide the three distributions that best fit the histogram.

After the modified EM converges, the parameters that maximize the likelihood function are then applied to segment brain image into three tissue classes. In this step we compute the membership probability with the estimated parameters. The initial brain volume is updated by adding the voxels, which are labeled as brain regions (WM, GM, and CSF) based on the value of membership probability. The process continues by checking every neighboring voxel of an already labeled one, until the left and right brain volumes remain unaltered. The outermost voxels of corresponding unaltered brain volumes provide the final left and right brain borders. The coordinates of each labeled voxels are stored to avoid double-checking of neighboring voxels during the brain volume updating.

The modified algorithm is summarized as follows.

1. Choose the number of Gaussian distributions and separate them based on the gradient-based method.
2. Select one Gaussian distribution in the split histogram.
3. Parameter initialization is as follows.
   E-step: estimate distribution over labels given a certain fixed model.
   M-step: choose new parameters for model to maximize expected  log-likelihood of observed data and hidden variables.
   Outputs: these are *μ*_*i*_ and *σ*_first-side_.
4. Assume the following.
  - The estimated mean is correct and the *σ*1 is the standard deviation of one side.
  - Standard deviation of the other side is *σ*2 = *r* · *σ*1.
5. *r* changes 10 percent at each calculation.
6. The defined threshold determines when calculation should be stopped.
7. Calculate error between the estimated distribution and real image histogram and obtain the optimal value of *r*.
8. If the error has reached threshold (an acceptable error that is defined in the first stage) then estimation is complete.
9. If error is more than threshold, estimation will be continued.
10. Continue steps (4) and (5) until the convergence of the sequence of parameters is reached.
11. Keep the estimated distribution and estimate the next separated distribution.
12. Compute the membership probability with the estimated parameters.
13. Assign each voxel to the *K*th class.

However, because of the intensity similarity between GM and CSF or CSF and WM and overlapping problem in the brain MR images the next improvement stage is required to have powerful segmentation. To have a robust and accurate segmentation in the next step we will extract some textural features from the image and finally use SVM to improve the classification process.

### 2.8. Feature Extraction

The goal of feature extraction is to reduce the original dataset by extracting the most important features. Choosing the optimal features has a strong effect on classification results. Image intensities are the most prominent features for image segmentation. Using intensity information as the only features in MR image is not sufficient due to several reasons.

- In some scans, the nonbrain voxels have a similar intensity to GM, WM, and CSF.
- The intensity of constructing brain tissues varies among different slices.
- In some slices, the intensity of different tissues is similar.

Therefore, we carried out texture analysis for describing texture of the images to have adequate features for accurate segmentation. We also extracted useful features such as first- and second-order texture information in this study to have an appropriate segmentation for all cases.

**Figure 6:**
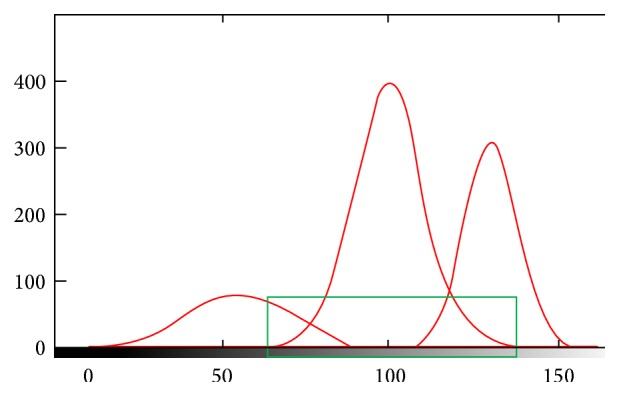
Target area or input data for 3D GLCM.

In the previous section voxel labeling is initially applied on each voxel of the brain using intensity information with some new ideas. Since overlapped voxels (voxels of brain borders) have two labels (GM, CSF or GM, WM) instead of one, to compensate for this problem, the overlapped voxels should be classified to identify which classes they exactly belong to. Thus, in this section to have robust and accurate brain segmentation, each overlapped voxel and its 18-connected neighbors are used as input for 3D statistical features extraction technique, which is an improvement stage. In other words the input of 3D GLCM is the target area in the rectangular region of interest [33] that is demonstrated in Figure 6.

One of the important issues in the field of image analysis is the question of how to determine the texture differences of complex images. These differences are often due to the relative emplacement of pixels of various intensities. One way to describe these differences in the spatial relationships of voxels is using a GLCM.

**Figure 7:**
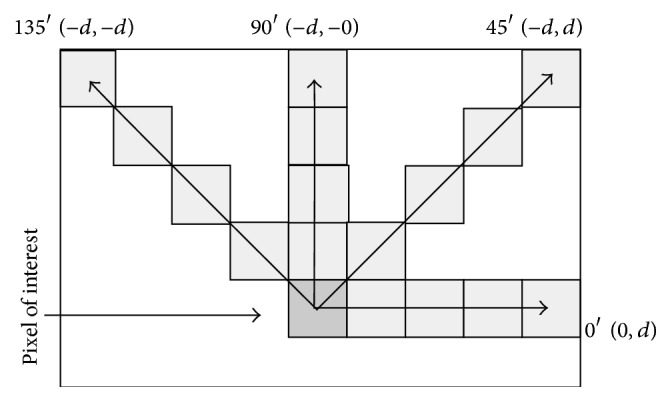
The spatial relationships of pixels, which are defined by the array of offsets, and *d* represents the distance from the pixel of interest.

The objective of this work is to generalize the concept of cooccurrence matrices to *n*-dimensional Euclidean spaces and to extract more features from the matrix. The GLCM matrix, defined as *G*_*d*_^*ϕ*^(*i*, *j*), is a square matrix (size *N*), where *N* is the total number of voxels in the window and (*i*, *j*) entry represents the number of cooccurrences of gray levels *i* and *j* for voxels separated at a distance *d* in direction Φ. In other words, the GLCM provides information on how often a gray level occurs at different directions. Usually, four directions are considered in the 2D case (Figure 7): Φ = 0°, Φ = 45°, Φ = 90°, and Φ = 135°, but in 3D images 13 directions are considered.

### 2.9. 3D GLCM

In this paper, we proposed 3D GLCM for feature extraction. Therefore, instead of square window (*W* × *W*), we considered cubes of size *W* × *W* × *W*. Selecting the window size is one of the main issues with this step, as it can define the discrimination capabilities of the extracted features. The choice of the window size plays an important role in the segmentation process. A small window decreases the computational burden and also enables resolution to capture the texture. Furthermore, large windows capture textural characteristics, but they increase the processing requirement and memory. Moreover, the smaller windows reduce the processing time and make the results more accurate and vice versa for bigger windows. This way, we chose 21 × 21 × 21 windows as a trade-off between resolution and performance.

GLCM computation can be generalized as

(9) Gdϕi,j=∑z=1Vz−dz∑y=1Vy−dy∑x=1Vx−dx1,if  (Qx,y,z=i)hI∧Qx+dx,y+dy,z+dz   =jQx+dx,y+dy,z+dz,0,otherwise, hhhhhhhhhhhhhhhhhhhhhhhhhhhhhi,j=1,…,N,

where *v* = (*v*_*x*_, *v*_*y*_, *v*_*z*_) is the position of the voxel, *N* is the number of gray levels present in the images or subimages considered for GLCM calculation, and *d* = (*d*_*x*_, *d*_*y*_, *d*_*z*_) is the distance in each direction.

The GLCM is a well-established tool for characterizing the spatial distribution, which includes second-order statistics of gray levels in an image. Second-order statistics are the texture of the image as they take into account the relationship among voxels in a window. An element at location (*i*, *j*) of the cooccurrence matrix signifies the joint probability density of the occurrence of gray levels in a specified direction Φ and specified distance *d* from each other. The 3D cooccurrence matrix stores the number of cooccurrences of pairs of gray levels *i* and *j*, which are separated by a distance *d* (in this study, *d* = 1,2,…, 5 voxels) in 13 directions of the voxel of interest (VOI). In this paper, for each distance (*d*) thirteen 3D cooccurrence matrix features were calculated from a sliding window (21 × 21 × 21) within the brain volume, such as; angular second moment, contrast, correlation, variance, inverse different moment, and so forth.

In addition two first-order texture features (mean and standard deviation of each feature) over the thirteen cooccurrence matrices (corresponding to 13 directions) are calculated, comprising a total of 26 GLCM-based features for each distance *d*. In total, 130 features were calculated per VOI.

### 2.10. Feature Selection

As presented in previous sections second-order (textural) and first-order and histogram-based features are extracted from the image. Since using all the features does not provide the best results the next priority is to choose the subset of features most likely to recognize one tissue class from another. The challenge is that even a modest GLCM method with 3D and 4*θ* values can create many more textural features than are suitable for the number of cases that will be subjected to classification. There are a number of techniques available for dimensionality reduction of features.

In this paper Stepwise Discriminant Analysis (SDA) that is a statistical approach is used to reduce the dimensions of the feature [33]. Discriminant Function Analysis undertakes the same task as multiple linear regressions by predicting the outcome. Multiple linear regression is limited to cases where the dependent variable on the *y*-axis is an interval variable. Thus, the combination of predictors will create the estimated mean numerical *Y* values for given values of weighted combinations of *X* values. Discriminant Analysis (DA) is an earlier alternative to logistic regression that is recently mostly used.

After feature selection step the subset of features has been used to analyze the images on real and simulated database and to provide a powerful segmentation using the last step (SVM). We used SVM classifier especially for brain margin classification and segmentation enhancement.

### 2.11. Segmentation Enhancement Using SVM

In this paper brain pattern identification and characterization is achieved by initially classifying the brain volume into three classes based on the extended EM method. Due to the existence of artifacts and overlapped regions in the histogram of brain images the extended EM method is not enough for accurate segmentation. Therefore a feature extraction and then SVM classification are performed to obtain satisfactory results. For images without artifact the SVM step does not change the segmentation results. The problem is that all MRI images have artifacts to some degree and due to existence of these artifacts an improvement stage is necessary. Moreover as mentioned before, in brain images especially in borders there are overlapped voxels in the histogram. The existence of these overlapped voxels is inevitable in MRI and they make the segmentation inaccurate.

In our case, most of the false positive and false negative of WM are clearly located in the brain borders. In particular false positive and false negative of WM in brain borders may have a large influence on the relatively small total volume of WM. This problem is also similar for gray matter and CSF. In this study a postprocessing step is applied to compensate this problem. In other words, in the target area of Figure 6, when two Gaussians join each other, false positive and negative reach the maximum value and in the tails of Gaussians the false positive and false negative are decreased. In these areas the problem is that each overlapped voxel has two labels instead of one label and this problem degrades the accuracy of algorithm. In this paper to solve this problem we used SVM classifier as a postprocessing step, which is also a well known method to border identification.

SVM is currently considered a state-of-the-art method to solve binary classification problems. Because of generalization ability, SVMs have experienced great success in different applications [3, 36]. Since the SVMs attempt to maximize the separation margin, the generalization performance does not drop considerably even when the training data is scarce. SVMs work well for classification of the objects, which are not linearly separable. These objects are mapped into a high-dimensional feature space through kernel transformation. We can also replace or combine SVM with other classifiers to have better segmentation results [37].

Specifically in this study a support vector machine classifier is employed as an enhancement stage for segmentation to assign a label to each overlapped VOI sample of each class. This method is also used to rank computed features from the extracted features.

SVM is a linear discriminate classifier, which was developed in statistical machine learning theory by Vapnik as a linear binary classifier. In this stage SVM classifiers are trained for each brain tissue based on the set of extracted features from the target area. Most features are extracted from nonoverlapped regions. In addition to improving the SVM training process, some features of overlapped voxels are also extracted. In this section support vectors are briefly described.

The SVM classifiers require a training step to define a separating hyperplane for the data in the feature space. These hyperplanes separate various tissue classes so that the margin between the classes is the maximum margin.

The appealing characteristic of SVM algorithms is that they offer the possibility to apply a kernel function for transforming the data into a higher-dimensional feature space (*K*(*xi*, *xj*) = *φ*(*xi*)^*T*^ *φ*(*xj*)). The kernel makes the data linearly separated with a maximum margin. For soft margin classification we used slack variables *ξi*. In this paper, to enable nonlinear decision functions, we used radial basis function (RBF) kernel for parameter selection of SVM classifier. Since the SVM algorithm is designed for two-class classification, to enable multiclass classification the classification is extended by one-against-the-others strategy. The SVM classifier assigns a label of brain tissue using extracted features. The features are extracted from a (21 × 21 × 21) VOI that is centered at the voxels being labeled. As mentioned in Section 2.8, the choice of the window size plays an important role in the classification process, as it may determine the discrimination capabilities of the extracted features.

In the SVM step, the sliding window moves along target area and labels the overlapped voxels in this region. SVM input involves overlapped and some nonoverlapped voxels in target area.

We performed the training process in two steps. In the first step, each subject is trained individually. To improve the training process we used overlapped and some nonoverlapped voxels as training data and overlapped voxels as test data. In the second step, we used all subjects to have an accurate and robust classifier. Because the problem is in the region of overlapped voxels and after the first step of segmentation, most of them have two labels, and we used these voxels for testing step. Finally in testing step voxels labeling is performed completely and each voxel belongs to one class.

In this section 12 subject of T1-weighted images of IBSR and 12 samples of BrainWeb datasets were applied to the training process. We used 8 subjects as training data and 4 remaining subjects to test the performance of the training process in each dataset. SVM training requires fixing the penalty term for misclassifications (*C*). In this series of experiments, the *C* is set to 100. With an appropriate selection of metric within the RBF kernel, the “leap” in implementation did not occur, as normally expected by using RBF kernels. *χ* ^2^ or Laplacian RBF kernels decrease the Gaussian RBF error rate from around 30% down to less than 10%. This improvement is not only due to the selection of the proper metric, but also due to the suitable generalization of SVMs. The SVM classifier was trained for a total of 10 000 samples per training brain image that were randomly selected from the provided brain mask.

## 3. Experimental Results and Discussion

In this study to evaluate the performance of proposed technique we accomplished two sets of experiments, one on simulated images and another on real data. Since in these cases the ground truth (anatomical model or expert segmentation) is available, it is feasible to have a quantitative evaluation of the performance of method under different conditions and compare the results with the other state-of-the-art methods. To evaluate the performance of proposed algorithm on real images we compared the result with expert segmented images from IBSR dataset and finally compared the result (*K* indexes) with the other state-of-the-art methods. The evaluation result is presented in the next section.

Reliability of data collection is a component of overall confidence in an algorithm accuracy. The importance of rater reliability lies in the fact that it demonstrates the extent to which the data collected in the research study are true demonstration of the variables measured. Measurement of the extent to which data raters assign the same value to the same variable is called interrater reliability [38]. Although there have been different techniques to measure interrater reliability, it is measured as percent agreement, computed as the number of agreement scores divided by the total number of scores [38].

For this reason, in some studies in the literature the standard Jaccard similarity index of images is calculated. This metric measures the similarities between the two sets such as *S*1 and *S*2 as the ratio of the amount of their intersection divided by the amount of their union using (10) [5]. Two sets of *S*1 and *S*2, indicating the created and gold standard segmentations, respectively. Consider

(10) $$\begin{array}{c} JS\left(S1,S2\right)=\frac{\left|S1\cap S2\right|}{\left|S1\cup S2\right|}*100\%. \end{array}$$

The other metric usually applied to compare the set similarity is Cohen's Kappa statistic or simply Kappa coefficient [39], which is defined as

(11) kS1,S2−S1∩S21/2S1+S2 =S1∩S2S1∪S2−1/2S1∖S2+S2∖S1∗100%.

The Kappa is one of the most frequently used statistics in the literature to test interrater reliability [38]. This metric demonstrates that this is a special case of the *k* index, suitable for evaluation of image segmentation algorithm [39]. *K* metric is larger than the Jaccard metric, except at 1 and 0. These metrics are related to each other by the function [40]

(12) $$\begin{array}{c} k=\frac{2J}{J+1}. \end{array}$$

Both metrics agree that 1 means the two sets are similar and that 0 means the two sets are dissimilar. For the purpose of comparison, the mentioned metrics are consistent. For example, an increase in the *k* index means an increase in the Jaccard metric [40]. In this paper, the Kappa coefficient is defined for both phantom and real datasets and the results are presented in the next sections.

### 3.1. Simulated Brain MRI

As the ground truth is an image that is not known for the real data, the proposed algorithm has been first evaluated on simulated images. Knowing the anatomical model (ground truth) we can have a quantitative evaluation of the performance of the different methods and also compare them.

**Figure 8:**
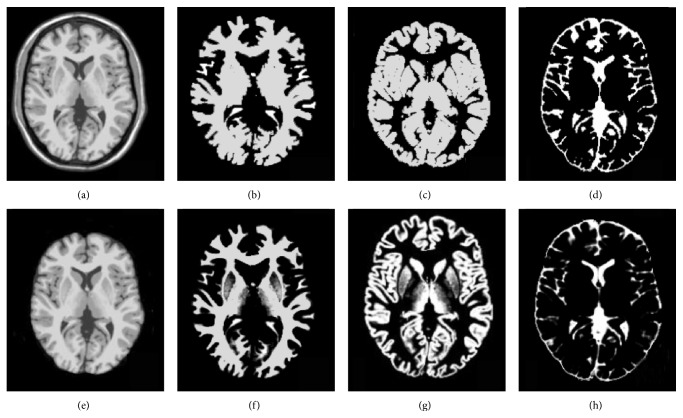
Results of segmentations on the BrainWeb images (a, e), synthetic image, and the extracted brain image, respectively (b, c, and d). Estimated WM, GM, and CSF, respectively (f, g, and h). The ground truth images of WM, GM, and CSF, respectively.

BrainWeb is a dataset providing simulated brain MR images for different acquisition parameters and acquisition modalities like T1 and T2 [40]. We applied the simulated MR scans of the head that are generated using the BrianWeb simulator (available from http://www.bic.mni.mcgill.ca/brainweb/) produced by the McConnell Brain Imaging Center at the Montreal Neurological Institute (MNI) [41]. Each MR image is provided with a ground truth that provides main tissue class labels for each voxel. For the technique, the considered BrainWeb images have been chosen with classical acquisition parameters (with respect to a standard brain MRI acquisition), namely, by considering T1-weighted images, with 1 mm resolution. The repetition time is equal to 18 ms and the echo time has been set to 10 ms. We used 18 synthetic volumes of 181∗217∗181. BrainWeb dataset has six degrees of noise contamination (i.e., pn0, pn1, pn3, pn5, pn7, and pn9) and the bias field (intensity nonuniformity) can be specified at three different levels (i.e., rf0, rf20, and rf40). For both our labeled results and the ground truth labeling, we generated three-class labeling (see Figure 8). The Kappa coefficient is computed for WM and GM tissues for each volume compared to ground truth [42].

To point out the contribution of the proposed method, we compared the proposed method with fuzzy and nonfuzzy methods with different Rician noise and 20% inhomogeneity as shown in Table 1. The results of each technique are averaged over the 18 volumes. The fuzzy methods are FCM [40] and NL-FCM [43] and nonfuzzy methods are EM, SPM 5 [4, 25, 44], HMC [45], and Fast [45]. SPM5, FCM, EM, and Fast are free available reference software for brain MRI segmentation. We carried out experiments to define the robustness to noise for the proposed technique with BrainWeb T1-weighted images.

In this study, as indicated in the first row of table, the *k* index of each method is defined by percent value. The percent value is computed by multiplying the numeric value of the *k* indexes by 100.

**Figure 9:**
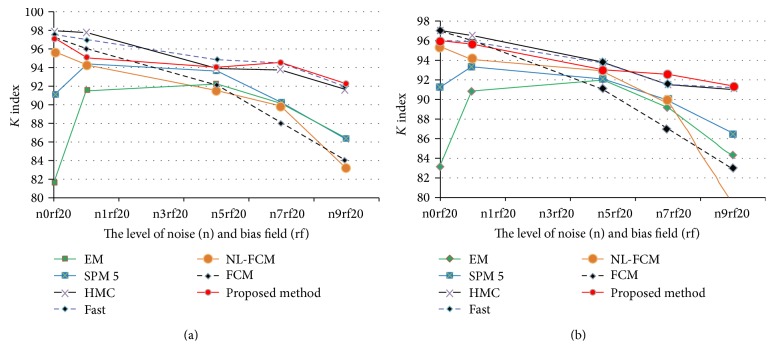
Average Kappa indexes of the simulated images. ((a) to (b)) The average Kappa indexes for WM segmentation. The average Kappa indexes for GM segmentation.

The average Kappa indexes over all 18 volumes in different methods for WM and GM are shown in Figure 9. As it can be observed from Table 1 and the graphs in Figure 9 the proposed method has notable superiority over the other methods specifically in image volumes that are seriously contaminated by random noise (i.e., pn0–pn9). For example, the presented Markov random field and Fast methods in the table are superior to our method in low-level noise, but in high-level noise the proposed method is superior. In addition Table 1 shows that the FCM based algorithms are not a reliable method in noisy image applications. The proposed method also presents satisfactory results in comparison with standard EM due to accurate demonstration of intensity distribution and using other features for segmentation. The average Kappa indexes in different levels of noise for WM are EM = 88.36, SPM 5 = 91.07, FCM = 91.4, HMC = 94.6, NL-FCM = 90.86, Fast = 94.72, and the proposed method = 94.46. The average Kappa indexes of GM segmentation are EM = 88.61, SPM 5 = 91.1, FCM = 91.3, HMC = 94.03, NL-FCM = 90.9, NL-Fast = 93.9, and the proposed method = 94.

### 3.2. Real Brain MRI

The proposed algorithm is also applied to real MR images, which are obtained from the Internet Brain Segmentation Repository (IBSR), which are available at http://www.nitrc.org/projects/ibsr. These brain image datasets and their manual segmentations were provided by the Center for Morphometric Analysis at Massachusetts General Hospital. This dataset of images is a set of 18 3D brain images with expert segmented volumes. The resolution of these images varies from 0.8 × 0.8 × 1.5 to 1.0 × 1.0 × 1.5 mm and they have a size of 256 × 256 × 128 voxels. The computation time of IBSR dataset is then globally similar to that one of a BrainWeb image.

Dealing with real data, we are faced with problems using Brain Surface Extractor (BSE) in separating the brain from nonbrain tissues. Indeed, some nonbrain regions still appear in the images, degrading the segmentation results. In order to handle this issue we used the atlas to separate nonbrain tissues.

**Figure 10:**
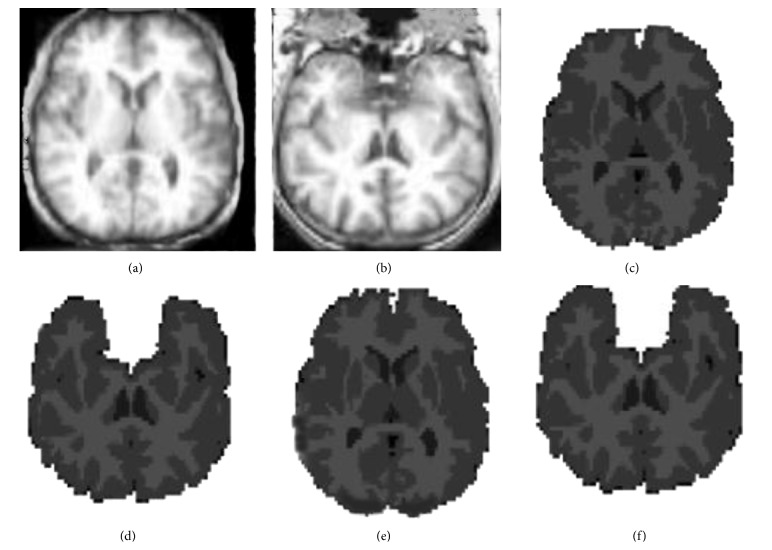
Proposed algorithm applied to IBSR database. Brain MRI slices of IBSR database. Expert segmentation. (e, f) Results of the segmentation of proposed method.

**Figure 11:**
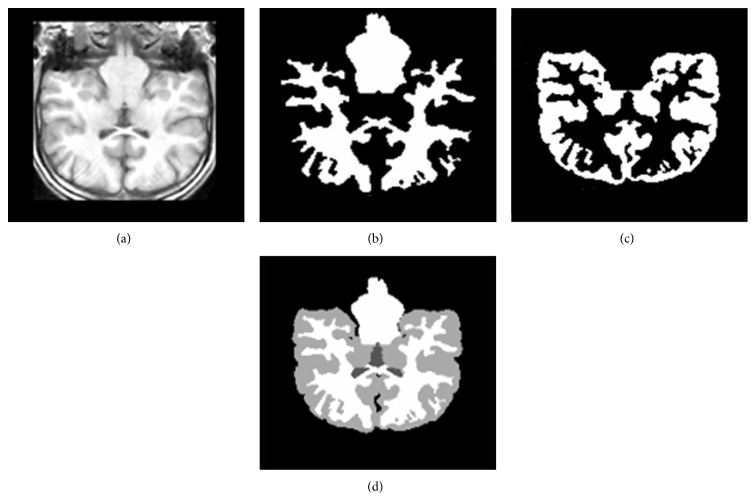
Results of segmentations on the T1-weighted IBSR image; (a) original image; (b) estimated WM image; (c) estimated GM image; (d) estimated CSF image.

For visual evaluation, two slices are selected from IBSR dataset and our method was applied on them. Figures 10(a) and 10(b) display two original selected slices. The manual segmented results are shown in Figures 10(c) and 10(d), respectively. The corresponding segmentation results achieved by our method are shown in Figures 10(e) and 10(f), respectively.  Figure 11 demonstrates the other slice of the original IBSR volume after brain segmentation.

Comparison with manual segmented images demonstrates that the proposed method outputs provide satisfactory results because the similarity between manual segmented and automatic segmented images could be observed (see Figures 10 and 11). For more analysis, our method is applied on all 18 real images of IBSR and to measure the similarities between the two sets we calculated *K* indexes of images and compared them with the other state-of-the-art methods. The results of our comparison are satisfactory based on the *K* index values (see Tables 1 and 2).

Since IBSR is generally used brain MR images for the validation of tissue segmentation, the results of the mentioned algorithm can be compared to those obtained by the other state-of-the-art techniques, particularly the following ones: hidden Markov chains (HMC) [43, 46], expectation-maximization (EM), statistical parametric mapping (SPM 5) [4, 45], Fuzzy *C*-Means (FCM), Nonlocal Fuzzy *C*-Means (NL-FCM) [45].

Based on these considerations, the overlap measures are computed for WM and GM and the average results obtained in the 18 cases are compared to the ones of these other techniques.

Since the brain images in the IBSR dataset are segmented only into pure tissue classes, our segmentation results are converted into three classes (WM, GM, and CSF). As the IBSR ground truth includes only internal CSF while our technique also defines sulcal CSF, we do not report results for CSF. The quantitative mean results are also presented in Table 2 while results for each method are depicted.

From the measures of Table 2, it appears that all the segmentation algorithms considered in these experiments provide approximately similar results for the WM. When considering the results, the use of the proposed method globally leads to better results than the other state-of-the-art techniques (in terms of both the mean value and standard deviation).

**Figure 12:**
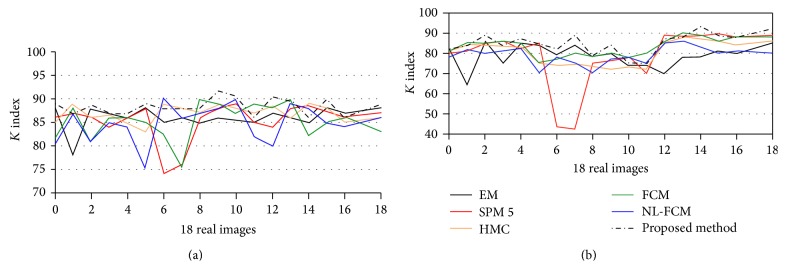
Kappa index calculated throughout the images in the IBSR database. ((a) to (b)) The Kappa indexes for WM segmentation. The Kappa indexes for GM segmentation.

Figure 12 presents the Kappa index for the 18 volumes from the IBSR database. The values of overlap measures in Figure 12 are based on published results and free available reference software for brain MRI segmentation.

The average Kappa index of WM segmentations is slightly improved in IBSR dataset. On the other hand the average Kappa indexes of GM segmentations suggest that the improvement is substantial. This variation may be attributed to the various spatial complexities of WM and GM. However, for both WM and GM, voxels of the same tissue class are connected to one another; GM tissue is inherently much more tortuous than WM tissue. Together with the fact that the standard Gaussian model does not precisely demonstrate the intensity distributions of real images, the proposed technique tends to improve the segmentation. In other words, the better performance of the proposed method over standard EM can be attributed to its accurate demonstration of intensity distribution. On average, however, the proposed technique still outperforms other competing techniques in classifying GM voxels. The average Kappa indexes of IBSR images for WM are EM = 86, SPM 5 = 85.3, FCM = 85.4, HMC = 86.91, NL-FCM = 84.83, and the proposed method = 87.20. The average Kappa indexes of GM segmentation are EM = 78.72, SPM 5 = 78.6, FCM = 83.16, HMC = 80.36, NL-FCM = 78.9, and the proposed method = 84.40. Furthermore, brain extraction step can cause differences in the final results of segmentation in terms of the Kappa index, as the number of voxels in the segmentation references can differ depending on the brain extraction algorithm.

**Figure 13:**
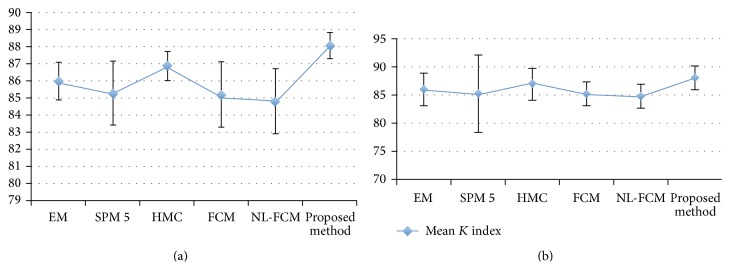
Mean and standard deviation of *K* index for segmentation methods in Figure 12. ((a) to (b)) WM graph, GM graph.

As statistical analysis we considered standard deviation and mean of *k* index for 18 real images and different methods. The robustness and accuracy of techniques could be evaluated by the amount of mean and standard deviation. In other words the larger mean leads to more accurate result and the smaller standard deviation leads to robustness (see Figure 13). In Figure 13 the vertical lines demonstrate the standard deviation and the blue graph indicates the mean overlap rate of different techniques for 18 real MRI. Figure 13 and Table 2 display that the proposed algorithm outperforms other competing methods.

In WM segmentation the mean overlap measure of our method is 88.35, which is 2% to 5% higher than other methods. In addition the standard deviation of *k* index of proposed method is 1.70, which is 1% to 4% less than other techniques. In addition, in terms of GM classification, the results of our algorithm are significantly better than WM segmentation.

Moreover, brain extraction step may cause differences in the results of brain classification in terms of the *K* index, as the number of voxels in the segmentation references may vary depending on the brain extraction method.

## 4. Conclusion

In this paper, we proposed a new automatic algorithmic framework for brain tissue segmentation using a novel combination of modified EM, 3D GLCM, and SVM.

Since using intensity information as the only feature in MR image is not sufficient to have a robust segmentation, in the proposed technique the spatial information and intensity information are used in different stages. The intensity information is used for the initial segmentation by EM based method. After feature extraction we used the target area from the first segmentation step to train SVM. Consequently to decrease the training and testing time of SVM and to have an accurate segmentation, we used location information as well as MRI intensity information as input features. The support vector machine classifier is employed to assign a label to each overlapped VOI sample of each class.

On the other hand, most statistical segmentation techniques in the literature have assumed that the intensity distribution of each tissue type is Gaussian distribution. However, the manual segmentation results provided by the IBSR dataset suggested that intensity distributions of brain tissues could vary asymmetrically. Thus an initial segmentation of the brain image into primitive regions is set by applying a modified EM method. In this step we assumed that the real brain MR images have asymmetric Gaussian distribution. The proposed EM algorithm overcomes the shortcoming of the standard EM technique using asymmetric Gaussians.

This step demonstrates a new method to overcome the problems with estimating the symmetric standard deviation of each Gaussian in the histogram. The experimental results indicate that the combination of the statistical and the machine learning based segmentation methods can enhance the overall segmentation performance, compared with each component individually. This is because the proposed method takes advantages of the classification ability of machine learning method in addition to the MR intensity and location information, which are consequential information to classify the brain in a 3D MRI into the multiple classes. In this paper to improve the overall segmentation performance different types of features are integrated, which are textural features, MR intensity, relationship with neighboring voxels, and voxel location. Robustness to noise and simplicity are two advantages of proposed framework. The results are independent of registration step and it makes our algorithm faster than other registration-based methods. In addition because our method is designed to run in Matlab, it is not platform-dependent and it can be run in both Linux and Windows operating systems.

In order to assess the proposed approach, it has been applied to brain tissue MR segmentation using real and simulated data, producing satisfactory results with respect to segmentation performance. The experimental results demonstrate that the integration of machine learning and statistical based segmentation techniques can improve the overall segmentation performance, in comparison with its individual components. This improvement is because the proposed technique takes advantage of the classification ability of machine learning algorithm in addition to the location and voxel intensity information, which are consequential information for brain MRI segmentation into the different classes.

Experiments on real data from the IBSR and synthetic images from BrainWeb have indicated that the proposed method achieves higher Kappa indexes compared with other methods currently in use. Incorporating spatial techniques such as 3D GLCM into the proposed approach could lead to interesting alternatives.

The proposed method not only preserves simplicity, but also has the potential to be generalized to multivariate versions adapted for segmentation applying multimodality images (e.g., T1, T2, and PD images). Experiments were first performed on different noise (up to 9% Rician noise) and 20% inhomogeneity BrainWeb MR images. These experiments show the precision and robustness of our method in the presence of different levels of noise and bias field. Additional experiments run on real MR images from the IBSR database have demonstrated that this method reliably extracts brain tissues with accuracy comparable to state-of-the-art techniques.

In terms of application, the proposed technique can be useful in the case of low contrast images (challenged by inherently low contrast tissue boundaries), for example, in the study of the developing human fetus. Extension of the proposed method for tumor and disease detection is the next challenging task for the future.

## Figures and Tables

**Table 1:** The Kappa index for the 18 volumes on the BrainWeb database with different Rician noise levels and 20% inhomogeneity.

Methods	White matter (%)					Grey matter (%)					
Noise level	0	1	5	7	9	0	1	3	5	7	9
EM	86.1	91.5	92.2	90.1	86.4	83.1	90.8	92.5	92	89.1	84.2
SPM 5	91.05	94.2	93.6	90.2	86.3	91.2	93.4	93.3	92.1	90	86.6
HMC	97.8	97.7	93.9	92.3	91.7	97	96.5	95.1	93.7	91.6	90.3
Fast	97.4	96.8	94.8	94.3	91.9	96	95.8	95.3	93.8	91.5	91.1
FCM	97	96	92	88	84	97	96	94	91	87	83
NL-FCM	95.6	94.2	91.5	89.8	83.2	95.4	94.1	93.8	92.9	89.9	79.3
Proposed method	97	95	94.9	94.4	92.2	95.9	95.7	95.3	93.8	92.1	91.2

**Table 2:** Mean and standard deviation of the Kappa index for different segmentation methods.

Methods	White matter (%)		Grey matter (%)	
	Mean	Standard deviation	Mean	Standard deviation
HMC	86.53	1.73	79.94	5.57
EM	85.87	2.27	78.94	5.68
SPM 5	85.27	5.52	78.7	13.98
FCM	85.6	3.81	83.21	4.03
NL-FCM	84.68	3.38	78.84	4.07
Proposed method	85.90	2.89	82.21	3.95
